# Supplementary material for: Inequality of opportunity in child nutrition in Pakistan
Source: PLoS One. 2025 Feb 21;20(2):e0318425. doi: 10.1371/journal.pone.0318425 (PMC11844883; doi:10.1371/journal.pone.0318425)
Supplement: Attachment — Submitted filename: Response to Reviewers.docx [file pone.0318425.s001.docx]

**The introduction is perfectly articulated and thus reflecting the nature of the IOP in Pakistan. However, this seems like enough data, information and literature has already been made on the topic. So, authors should provide clear research gaps and justify their current research questions to indicate the rationale of the new analysis.**

While national-level studies on child stunting are abundant, there is limited research focusing on the sub-national or provincial disparities in Pakistan. Many studies tend to aggregate the data at the national level, masking regional inequalities that may have distinct drivers and implications. Moreover, majority of previous stunting studies focus on inequality in outcomes (such as the prevalence of stunting), but relatively a few apply the inequality of opportunity framework for deeper understanding of structural inequalities, which distinguishes between factors children cannot control (e.g., parental education, socioeconomic background) and those they can (e.g., individual efforts). Further, there is a dearth of literature in unravelling the contribution of various factors in overall inequality in stunting via decomposition techniques. Additionally, very few studies in Pakistan have explored the association between access to water and sanitation and stunting. Hence, this study was undertaken to abridge the gap in the previous research using a more rigorous and nuanced approach

"The introduction part has been revised to incorporate the rationale clearly"

**Though the data used for this study was curated from the PDHS, however, it would be recommended to introduce more about how data was collected, sampled and curated in for the very primary by the NIPS, and how that was curated for the current study.**

"The methodology section has been revised to include the data collection and sampling strategy"

**Authors are recommended to provide justifications for the variables they used for the analysis of IOP.**

"The rationale of the variables used in the analysis of the IOP has been included in the revised version of the manuscript"

**Including some other similar, and highly relevant variables like food security, age (though it was base of of the sample selection, the age group (months), access to healthcare, would enhance the clarity of the hypothesis and study outcomes.**

The study is based on anthropometric measures which indicates nutritional insecurity among children. The age of the child and sex have been included in the analysis. The data on food security for children is not available in the PDHS, nor the study aims to look at the food security among households and link it up with nutritional insecurity among children.

**Other than 'stunting', the wasting, underweight and under-nutrient are some other indicators for measuring the IOP. But in this paper, authors only focused on stunting. Is there any justification? why not others?**

Rationale of using stunting:

Firstly, Pakistan has one of the highest stunting rates in the world. According to the Pakistan Demographic and Health Survey (PDHS) 2017-18, approximately 38% of children under five in Pakistan are stunted, compared to lower rates for wasting (7%) and underweight (23%).

Secondly, Stunting reflects chronic malnutrition, which results from long-term deprivation of adequate nutrition, healthcare, and living conditions during critical growth periods in early childhood. It signifies more persistent, structural inequalities. Unlike wasting (which reflects acute malnutrition) or underweight (which could indicate both acute and chronic malnutrition), stunting captures long-term inequality in opportunity that children experience due to socioeconomic and environmental factors. Stunting reflects *structural disadvantages that accumulate over time*, such as poor maternal health, poverty, and lack of access to quality healthcare and sanitation, making it more suitable for examining the deep-rooted inequalities in Pakistan.

Thirdly, In the context of IOp, stunting reflects inequalities in circumstances beyond a child’s control, such as parental education, socioeconomic status, geographic location, and access to healthcare and clean water. Because stunting is tied to long-term deprivation, it is a more appropriate marker for structural inequality compared to wasting or underweight, which may fluctuate more with temporary crises.Stunting often correlates with disparities in rural vs. urban areas, regional inequalities, and educational attainment of parents. For example, children from poorer provinces (such as Balochistan) or from lower socioeconomic backgrounds tend to exhibit higher stunting rates, making it a valuable metric for exploring IOp. Stunting is a comprehensive indicator of deep-seated inequality, capturing the multi-dimensional nature of disadvantage in Pakistan, from health and nutrition to education and socioeconomic conditions.

Fourthly, Stunting is strongly associated with impaired cognitive development, which affects a child’s educational outcomes, earning potential, and productivity later in life. Wasting, while serious, tends to have more short-term implications (i.e., increased risk of mortality due to acute malnutrition). In the context of inequality of opportunity, stunting has far-reaching impacts on future human capital development. Children who are stunted are less likely to reach their full potential, exacerbating intergenerational poverty and limiting social mobility. The long-term effects of stunting on cognitive development and economic outcomes make it a critical indicator for understanding inequality of opportunity in Pakistan. Tackling stunting addresses more than just physical health; it also has implications for educational achievement and economic inequality.

**Prevalence of IOP is apparently seen higher among the urban children, however, in general sense, the urban population are highly privileged and opportunist in terms of access to services, healthcare facilities and other economic opportunities in urban sprawl in comparison to the rural. Could clearly clarify why this is nature?**

The higher inequality of opportunity in child stunting among urban children in Pakistan is driven by several factors:

1. The existence of urban slums and informal settlements where living conditions are poor and access to services is extremely limited.

2. Wider socioeconomic disparities within urban areas, creating stark inequalities between the rich and the poor.

3. The vulnerability of migrant families and the lack of support systems for the urban poor.

4.  Fragmented access to services and healthcare, where the poorest urban residents often face the greatest challenges in accessing quality care.

5.  The high cost of living in urban areas, contributing to food insecurity and malnutrition among the urban poor.

6.  Environmental hazards and poor sanitation in urban slums, leading to higher rates of disease and malnutrition.

These factors combine to make urban inequality of opportunity more pronounced, particularly for children living in poverty within urban areas, despite the general assumption that urban environments are more privileged. This paradox highlights the importance of addressing intra-urban disparities to reduce inequality of opportunity in child health outcomes like stunting.

**Would this paper suggest some pragmatic recommendations like -improving access to healthcare and nutrition services, enhancing social safety nets programs, and promotion of accessibility to the economic inclusions for the segments - based on the study outcomes?**

*"The manuscript has been revised to include policy recommendations"*
